# Design Space Exploration of a Sparse MobileNetV2 Using High-Level Synthesis and Sparse Matrix Techniques on FPGAs

**DOI:** 10.3390/s22124318

**Published:** 2022-06-07

**Authors:** Antonios Tragoudaras, Pavlos Stoikos, Konstantinos Fanaras, Athanasios Tziouvaras, George Floros, Georgios Dimitriou, Kostas Kolomvatsos, Georgios Stamoulis

**Affiliations:** 1 Department of Electrical and Computer Engineering, University of Thessaly, 383 34 Volos, Greece; antragoudaras@e-ce.uth.gr (A.T.); pastoikos@e-ce.uth.gr (P.S.); kofanaras@e-ce.uth.gr (K.F.); attziouv@e-ce.uth.gr (A.T.); georges@e-ce.uth.gr (G.S.); 2 Department of Informatics and Telecommunications, University of Thessaly, 35 100 Lamia, Greece; dimitriu@uth.gr (G.D.); kostasks@uth.gr (K.K.)

**Keywords:** deep neural networks, High-Level Synthesis, hardware accelerators, FPGAs, sparse neural networks

## Abstract

Convolution Neural Networks (CNNs) are gaining ground in deep learning and Artificial Intelligence (AI) domains, and they can benefit from rapid prototyping in order to produce efficient and low-power hardware designs. The inference process of a Deep Neural Network (DNN) is considered a computationally intensive process that requires hardware accelerators to operate in real-world scenarios due to the low latency requirements of real-time applications. As a result, High-Level Synthesis (HLS) tools are gaining popularity since they provide attractive ways to reduce design time complexity directly in register transfer level (RTL). In this paper, we implement a MobileNetV2 model using a state-of-the-art HLS tool in order to conduct a design space exploration and to provide insights on complex hardware designs which are tailored for DNN inference. Our goal is to combine design methodologies with sparsification techniques to produce hardware accelerators that achieve comparable error metrics within the same order of magnitude with the corresponding state-of-the-art systems while also significantly reducing the inference latency and resource utilization. Toward this end, we apply sparse matrix techniques on a MobileNetV2 model for efficient data representation, and we evaluate our designs in two different weight pruning approaches. Experimental results are evaluated with respect to the CIFAR-10 data set using several different design methodologies in order to fully explore their effects on the performance of the model under examination.

## 1. Introduction

DNN training and inference operations are considered very demanding in terms of computation intensity and power consumption. As a result, such operations are frequently mapped on GPU systems in order to accelerate the execution speed of the corresponding DNN models. Despite the fact that high-performance computing seems to tackle, at least up to a certain point, the computational challenges of the DNNs, low-power architectures are having trouble balancing the performance-to-power trade-offs of the neural networks. With the emergence of Internet of Things (IoT) networks, low-power designs are pushed to the spotlight in both academia and industry domains due to the low-energy requirements of the IoT devices.

However, the hardware implementation of DNNs requires the mapping of several complex data processing algorithms on a field-programmable gate array (FPGA), starting by developing the circuitry in the RTL description in order to finalize the design process. Thus, developing DNNs directly in an RTL description is a very demanding task where the design time can take several weeks. Nowadays, there exists a wealth of HLS tools that enable developers to perform design space exploration on large hardware design spaces without requiring any development of the RTL modules. In this sense, HLS tools allow users to minimize the development time and efficiently explore the design space that corresponds to the application under examination. As a result, designers can identify potential design problems and examine the relevant configurations that can achieve the desired trade-offs between resource utilization and performance [1].

Due to the large amount of data processing operations contained within the inference level, researchers employ sparsification techniques along with dedicated hardware design methodologies. Pruning DNN models has proven to be an efficient approach to reduce their computation complexity, while acceptable levels of accuracy can be maintained [2]. Moreover, along with the reduction in computational complexity, there is a significant decrease in memory requirements, which are a critical specification of IoT devices. In this way, techniques for sparse data representation, such as compressed column and compressed row, are frequently employed in order to improve the memory footprint of the executing applications.

Under this premise, this work focuses on the acceleration of the DNN inference operation in IoT devices with low-power constrains using a state-of-the-art HLS tool. More specifically, we investigate how HLS tools can be utilized in order to perform the design space exploration of a sparsified MobileNetV2 under different configurations and provide meaningful insights regarding the developed designs. Furthermore, we present several design approaches, both in algorithmic and at the hardware level, that can generate the proposed sparsified DNN, such as compression techniques, and clock frequencies. Furthermore, we opt for compressed sparse row and compressed sparse column sparsification techniques, mainly due their popularity in the existing literature and their applicability in real-world applications. Such representations are considered one of the simplest yet very effective and well-established compression types.

Finally, we demonstrate that the proposed MobileNetV2 designs, produced by the Xlinx HLS tools, can achieve performance levels that are comparable with designs developed in hardware description languages by evaluating our hardware using the CIFAR-10 data set.

The rest of this article is organized as follows. Section 2 presents the previous work on existing DNN hardware accelerator techniques developed for the inference process. Section 3 presents the theoretical background of the MobileNetV2 computational complexity and elaborates on the employed sparsification techniques. Section 4 presents our main contributions on the application of a state-of-the-art HLS tool for the efficient design space exploration of the DNN model. Section 5 presents our experimental results, which are followed by conclusions in Section 6.

## 2. Related Work

Nowadays, DNNs are being applied in a wide range of recognition applications such as image and video classification. As DNNs are used in even more demanding tasks, in order to solve complex real world problems, their computational and storage requirements continue to increase. While DNNs have been successfully mapped on GPUs and CPUs, their mapping to platforms with limited resources remains a challenging task. Even if application-specific integrated circuits (ASICs) can achieve high throughput and are energy efficient, the design cycle of such devices is a strenuous process. Moreover, ASICs lack reconfigurability, which is a very desired attribute. These issues have made FPGAs and HLS tools very promising for designing complex DNNs designs and efficiently mapping them into these real-wrold platforms. In this section, we briefly describe some previous works in the area of DNN hardware accelerator techniques for ASICs and FPGAs.

In the ASIC domain, one of the most prominent hardware designs for DNN acceleration is the systolic array architecture. Such designs consist of a number of functional units which are interconnected through a mesh-like network and are dedicated to execute DNN operations. The emergence of these architectures has led to the development of google’s tensor processing unit (TPU) [3], which is a systolic processor capable of executing both inference and DNN training operations. Application-specific designs such as TPU depict a significant performance increase and energy reduction compared with standard CPU and GPU architectures. Similarly, previous work in [4] proposes a co-processor design tailored for DNN inference. Such an approach utilizes systolic arrays to accelerate the multiply-and-accumulate (MAC) operations and employs special purpose registers to store the model weights or the input data. In [5], the authors propose the deployment of a systolic array architecture for both DNN inference and DNN training. In this work, researchers exploit the dataflow parallelism of the DNN operations while also employing data reuse co-optimization techniques. Previous work in [6] utilizes a systolic array architecture for DNN inference by accelerating dense linear algebra operations after compressing the trained DNN model. In [7], authors also consider the efficiency of a SoC DNN accelerator for sparse models. In order to avoid the functional unit under-utilization issue, they develop a technique that exploits the slack caused by model compression to increase the network throughput. Finally, in [8], researchers develop Eyeriss v2, a DNN inference accelerator that is tailored for energy-constrained platforms that target sparse DNN models. The Eyeriss v2 architecture employs a flexible on-chip network that adapts to the different amounts of data reuse and bandwidth requirements thus, improving the utilization of the computation resources.

In contrast with ASIC, there are several optimization techniques regarding the DNN acceleration, that exploit the DSP units of Xlinx and FPGA platforms. A DSP unit can efficiently implement the MAC operations. In [9], the authors propose an automated Platform for Accelerator CreatIon (PLACID) to develop high-throughput hardware accelerators. In addition, the authors in [10] exploit the inherent parallelism of DNNs to reduce the resource utilization and power consumption of the system. These highly regular parallel computations are also explored in [11]. Another popular way to exploit the FPGA capabilities is to convert DNN into binarized neural networks (BNN). Practically, the MAC operations are replaced by XNOR computations that utilize one-bit operands only. In [12], the authors propose a complete framework for the efficient mapping of binarized neural networks to hardware. Moreover, in [13], the authors propose an efficient mapping approach of a BNN in C++ to Verilog using the Xlinx HLS tool.

Although previous works deliver promising results, they do not tend to explore or evaluate the proposed designs with respect to various architectural choices. The works in [14,15] bear a small resemblance to our approach, since they perform a design space exploration for mapping DNNs into FPGAs. However, these works do not explore pruning methodologies and sparse matrix techniques that have come to forefront in order handle the tremendous amount of increased computations. Clearly, the potential of sparse DNNs that comes from pruning techniques [2] has not yet been sufficiently explored. In this work, the proposed methodology explores several algorithmic and hardware aspects of a sparse MobileNetV2 to efficiently map the model into a targeted FPGA.

## 3. Background

### 3.1. MobileNetV2 Computational Complexity

**Figure 1:**
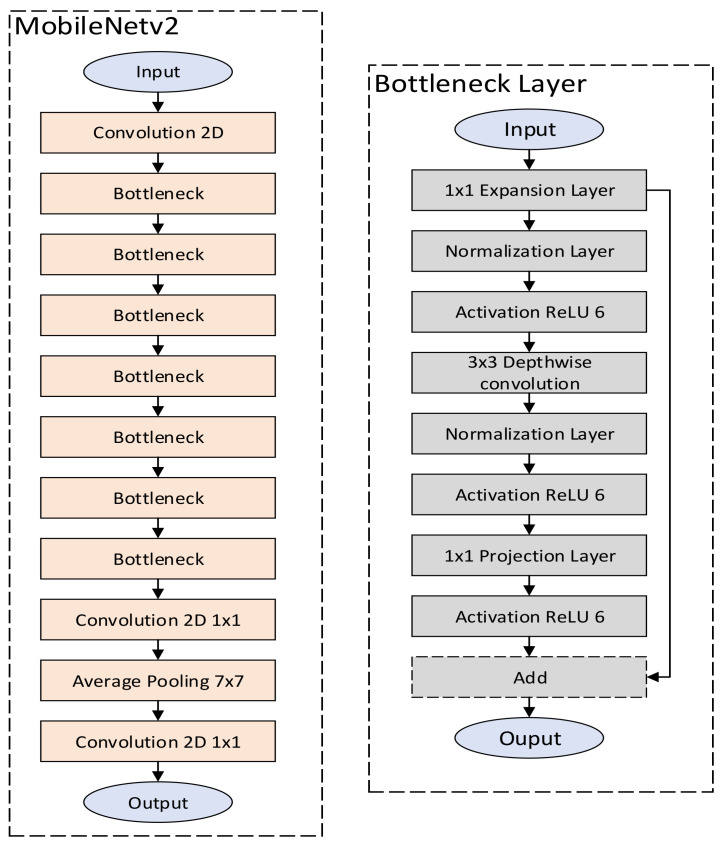
The architecture of MobileNetV2 DNN.

MobileNetV2 is a lightweight DNN architecture [16] which was introduced by google and targets mobile platforms and processors with low processing capabilities. MobileNetV2 is one of the best-performing DNN [17] in terms of accuracy and model size, and thus, it is considered ideal for IoT applications. Figure 1 below depicts the MobileNetV2 DNN model architecture. It consists of 2D convolutional, bottleneck and pooling layers. The bottleneck layer, in turn, consists of expansion, normalization, activation, addition and 3 × 3 depth-wise convolution sub-layers. The purpose of the expansion operation is to expand the data space in terms of increasing the number of channels of the input data by a factor that is defined by the model hyperparameters. On the contrary, the projection operations decrease the amount of channels of the input data and thus, they shrink the data space. The normalization, pooling, convolution and activation layers employ the corresponding operations that are necessary for the DNN training and inference process.

The DNN training procedure requires a forward pass of the data through the neural network, which is followed by a backward propagation operation which fine-tunes the model weights according to the value of the loss function. On the contrary, the DNN inference does not include any weight tuning process of the model, and thus, it requires the input data to be propagated through the network once. As a result, the DNN inference process is considered less cost demanding in terms of computational requirements and energy consumption when compared with the DNN training. More specifically, the inference process of a trained MobileNetV2 model requires 13 million MAC operations and contains 4.3 million parameters. As a result, the design and implementation of the MAC operation plays a critical role on the execution time of the MobileNetV2 inference.

### 3.2. Sparse Neural Networks

Nowadays, DNNs have shown remarkable success at the cost of a huge amount of parameters and MAC computations. However, methods based on weight pruning have been established as an attractive approach to reduce the computation and memory requirements of DNN models without sacrificing significant accuracy levels [2]. In this work, we employ a structured pruning technique based on [18] in two modes. Firstly, a conservative threshold is employed in order to maintain the accuracy of the MobileNetV2 in high degrees. Secondly, a more aggressive pruning approach is utilized in order to be compared with respect to the baseline models and the conservative approach. It is worth mentioning that unlike unstructured pruning methods, structured pruning techniques are usually more hardware-friendly methodologies, since they present higher regularity, while also they can achieve comparable pruning rates. We evaluate all the approaches using compressed column and row formats in order to minimize the memory requirements and efficiently accelerate the data utilization rate for the MobileNetV2 operations. Within each approach, hardware kernels in the same design are encoded with similar compressed formats. In this way, a common compression schema is used, reducing the irregularity of sparse weights. The complete sparse wise dataflow and the proposed architecture is depicted in the next section.

## 4. Design of Neural Network Hardware Accelerator

### 4.1. System Architecture

**Figure 2:**
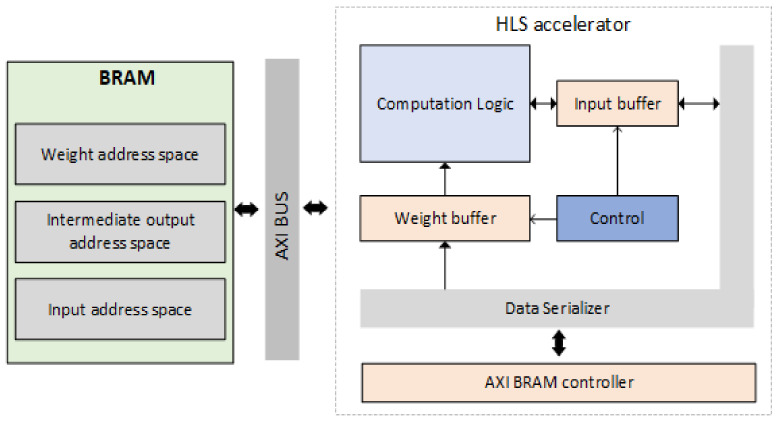
High-level architecture diagram of the MobileNetV2 accelerator.

The overview of the system architecture is illustrated in Figure 2. Our design employs a BRAM with dedicated address spaces for weight, input data and intermediate output, correspondingly. The BRAM-accelerator communication is performed through an AXI bus and is managed by an instantiated AXI BRAM controller that is capable of performing the virtual-to-physical address translation. Regarding the HLS accelerator, we deploy a data serializer module that reads and decodes the data representation format of sparse matrices. Data serializer transforms the compressed data to a format that is usable by the computational logic and vice versa. Furthermore, we implement two separate data structures, i.e., weight and input buffers, that temporarily store the weights and outputs of the computation logic. Such buffers manage to lower the amount of BRAM access operation during the inference process, since they exploit the data re-usability of the model layers. As a result, the data movement between the BRAM side and the accelerator module is reduced, thus saving energy consumption and increasing the throughput of the circuit. The buffer read/write operations are managed by a control unit that designates which data should be stored to the corresponding buffers. The computation logic module is responsible for conducting the arithmetic operations (multiply-and-accumulate) which are required for the inference process. It is instantiated through an HLS approach and utilizes inputs from the input and weight buffers while its results are stored back to the input buffer.

### 4.2. HLS Accelerator

The HLS accelerator should be able to perform the three convolution operations which consist of the building blocks of the MobileNetV2 model, i.e., standard convolution, depth-wise convolution and point-wise convolution. Generally, there are two types of approaches when designing an application-specific integrated circuit:

- Flexible designs. In such scenarios, the design will be able to execute operations with variable operand size (e.g., 6-bit, 12-bit or 32-bit wide) by leveraging a complex interconnection network, which propagates the intermediate results to the corresponding functional units. In our case, a flexible design choice would guarantee that our accelerator could execute all three convolution operations within one hardware module.
- Non-flexible designs. Non-flexible designs are implemented to execute a specific task without being able to adapt to support additional operations or variable operand width. In our case, a non-flexible design choice would require three separate modules, each one dedicated to one convolution operation.

In this work, we opt for a non-flexible design due to the high operating cost of flexible accelerators in terms of power consumption. Despite their flexibility, such designs utilize interconnection fabrics which require a significantly larger amount of power to operate. On the contrary, non-flexible designs employ simpler interconnects but they utilize more area, since hardware modules are dedicated to certain execution tasks. In this sense, we opt to trade higher area requirements with smaller power consumption.

Algorithm 1 depicts the HLS pseudocode of the accelerator module. The *computational logic* is charged with the execution of convolution operations and its outputs are either discarded or stored in the *input buffer*. Such a decision is taken by the *control unit* which manages the write rights of both *weight* and *input buffers*. The *control unit* identifies data dependencies between consecutive convolution operations and exploits data re-usability if it is detected. In any other case, it generates the necessary control signals to reroute data from the *Data serializer* to the *buffer* structures. *The Data serializer* is responsible for translating compressed data to a format that is usable by the *computational logic*. Such data are fed to the *Data serializer* by the *AXI BRAM controller*, which manages the HLS-BRAM communication.
**Algorithm 1** HLS pseudocode of the accelerator.FloatBuffer WeightBuf [WBUF_SIZE]FloatBuffer InputBuf [IBUF_SIZE]  **for** each convolution **do**  output = computation_logic(WeightBuf, InputBuf)   raw_data[weights] = BramCntrl(weight_addr, mem_out)  raw_data[inputs] = BramCntrl(input_addr, mem_out)   **if** compression_type == CSC **then**   serialized_data = Data_Serializer(raw_data, CSC)  **else if** compression_type == CSR **then**   serialized_data = Data_Serializer(raw_data, CSR)  **else**   serialized_data = raw_data  **end if**   WeightBuf_write_enable = controller(WeightBuf);  InputBuf_write_enable = controller(InputBuf);   **if** WeightBuf_write_enable == True **then**   **for** i=0; i<WBUF_SIZE; i++ **do**    WeightBuf[i] = serialized_data[weights][i]   **end for**  **else**   WeightBuf[i] = WeightBuf[i]  **end if**   **if** InputBuf_write_enable == True **then**   **for** i=0; i<IBUF_SIZE; i++ **do**    InputBuf[i] = output[i]   **end for**  **else**   InputBuf[i] =serialized_data[Inputs]  **end if****end for**

Regarding the implementation of the *computational logic*, we deploy separate HLS accelerators for each MobileNetV2 layer type, i.e., standard, depth-wise and point-wise convolution. Each accelerator is specialized in accelerating one convolution type only and utilizes the weight, input and output buffers for I/O operations. Below, we discuss the details of each layer type, and we provide the implementation strategy for the corresponding accelerators.

The SC can be described under the following equation:

(1) $$y=f\left(\sum_{k=0}^{C}\sum_{i=0}^{Ht}\sum_{j=0}^{Wt}\left(X\left[k\right]\left[i\right]\left[j\right]*W\left[k\right]\left[i\right]\left[j\right]\right)+B\right)$$

where *y* is the output matrix of the convolutional layer, *f* is the activation function of each neuron, *C* is the amount of weight channels, Ht is the height of the weight matrix, Wt is the width of the weight matrix, *X* is the matrix of the layer input, *W* is the matrix of the weight values and *B* is the layer bias. Generally, the SC can be viewed as a dot product of input matrix and the weight matrix adjusted by an activation function, which normalizes the output within a numerical range limit. In our approach, we employ the *Wbuf* and *Ibuf* structures to temporarily store the weight and input data correspondingly, and we proceed in calculating their dot product. The output is stored on the local buffer *Obuf* in order to reduce memory load/store operations, since it is used as input for the next layer.

The PW can be described using the following equation:

(2) $$y=f\left(\sum_{k=0}^{C}\left(X\left[C\right]*W\right)+B\right)$$

PW convolutions can be considered as a special case of SC where the weight (W) size is 1 (1 × 1 × 1 dimensions), and thus, the computational complexity of the PW is significantly reduced. Thus, a PW convolution utilizes a scalar weight value (referred to as a “point”) to perform the convolution operation with the whole sequence of input data. As a result, PW is used to combine the outputs created by previous layers without expanding the dimensions of the input matrix. Similarly to SC, we utilize the *Wbuf*, *Ibuf* and *Obuf* buffers to minimize BRAM access operations.

The equation that describes the DW operation for each weight channel can be written as follows:

(3) $$y=f\left(\sum_{i=0}^{Ht}\sum_{j=0}^{Wt}\left(X\left[i\right]\left[j\right]*W\left[i\right]\left[j\right]\right)+B\right)$$

DW, also called depth-wise separable convolution, separates the weights’ depth dimensions by performing a standard convolution operation for each channel separately. Country to the SC approach where weight channels are included in the SC operation, DW performs a SC for each channel and then concatenates the outputs into one multi-dimensional matrix.

In this work, we explore how the parameter space affects the system performance in terms of inference (test) accuracy, layer errors, model size, inference latency, area and power requirements. Our approach is focused on such aspects since we are considering the real-time performance of a DNN in real-world applications. More specifically, we provide insights regarding the following parameter space.

- **Architectural support.** We provide a simple yet effective hardware architecture to accelerate the three convolution types that reside within the MobileNetV2 model. We also deploy data buffers to reduce the BRAM–accelerator communication overhead. By employing such an approach, we attempt to explore whether hardware accelerators (in HLS) are capable of supporting real-time DNN applications.
- **Model compression.** We utilize a well-established model compression scheme to analyze its efficacy in DNN model size and inference latency.
- **Pruning approach.** We implement a conservative and an aggressive pruning mechanism to study their effects on the model performance.
- **Sparse data storage.** For the efficient storage of the sparse MobileNetV2, we deployed representations that exploit the sparsity of the matrices. As a result, we perform all data storage using sparse data structures, i.e., Compressed Sparse Row and Compressed Sparse Column (CSR/C). More particularly, these two approaches leverage the high amount of zero entries and eliminate them by replacing the matrix with a three-vector representation scheme. For example, the CSR approach scans the non-zero elements of the initial matrix in row-major order and stores them in a vector. In parallel, it creates two additional vectors, where the first stores the respective column indices and the second one stores pointers that designate where the new row begins.
- **Clock frequency.** We deploy designs with two different clock frequencies to examine their effects in the inference latency and power consumption of the system.

## 5. Evaluation

### 5.1. Experimental Setup

In order to perform the evaluation process, we employ a pre-trained MobileNetV2 DNN [16] model. The model, which is implemented in Pytorch, is depicted in Table 1 and consists of 10 standard convolution layers, 9 depth-wise convolution layers and 9 point-wise convolution layers, resulting in a total of 28 intense computation layers. The DNN also includes a number of expansion, pooling and activation layers as discussed in Section 3, but they contribute in a trivial way to the overall computation complexity, which is measured at 13 MFLOPs.

For the inference process, we use the CIFAR-10 data set [19], which is a collection of 60,000 images in 10 classes, with 6000 images per class. The images are segregated in two sets: a train set, which is used for the DNN training process and contains 50,000 images and a test set, which is used for the inference operation, with 10,000 images.

Table 2 depicts the parameters of the individual designs we have implemented in order to evaluate our approach. We have opted to employ 14 different designs to explore the effects of clock frequency, compression type and pruning techniques on the performance of the corresponding model. Below, we provide a list of individual parameters as well as a characterization of their distinguishable properties.

- Compression type: This parameter refers to the sparsification technique that is employed by the corresponding design. In this work, we initially used *compressed sparse row*, *compressed sparse column* and *no compression* formats. Since the evaluation results for the MobileNetV2 indicated that the *compressed sparse row* and *compressed sparse column* formats depicted similar characteristics in terms of accuracy, compression rate and power, we opt to use the naming convection *CSR/C* to note whether the design has undergone any compression process.
- Frequency: It specifies the core clock frequency of the implemented design. We employ two types of clocks: 50 MHz and 100 MHz.
- Pruning layers: Pruning refers to a collection of techniques that aim to reduce the amount of weights and eliminate neuron connection in order to minimize the computational complexity of the model. The *pruning layers* parameter specifies which layers are included in the pruning process. To this end, we opt for a *None* approach that does not utilize any pruning optimizations, a *Standard convolution* approach that applies pruning on the convolutional layers, a *Depth-wise convolution* technique that prunes only the layers associated with depth-wise convolution operations and a *Standard and Depth-wise convolution* methodology that prunes both standard and depth-wise convolution layers.We have decided not include any pruning process within the point-wise convolutional layers, since the amount of parameters in such layers is trivial compared to the rest of the model.
- Pruning approach: The pruning approach specifies what type of methodology is applied during the pruning process. Since there are a lot of different ways to prune a neural network, we note the techniques used within this work as *Conservative* and *Aggressive*. A *conservative* pruning approach eliminates the model weights that contribute less to the output of the network; thus, it removes weights that associate with low numerical values. In this work, we consider a numerical value as in [18], which indicates the employed pruning rate. The pruning rate for the *conservative* approach is set to 30%. On the contrary, an *aggressive* pruning approach utilizes a higher pruning rate, i.e., 65% to remove model weights. Thus, we expect the *aggressive* pruning to result in more weight removals compared to the *conservative* approach.

Furthermore, we implement two more designs, namely *BASE-50* and *BASE-100*, which are used as baseline implementations. The *BASE-50* and *BASE-100* do not employ any compression or pruning techniques, and their clock frequencies are tuned to 50 MHz and 100 MHz, respectively.

Regarding the evaluation platform, we opt for a ZedBoard Zynq-7000 ARM FPGA which incorporates a dual-core ARM Cortex-A9 processor with 512 MB DDR3 and 256 MB Quad-SPI Flash memory. For the evaluation process, we utilize the on-board USB-JTAG communication interface of the FPGA.

### 5.2. Evaluation Methodology

**Figure 3:**
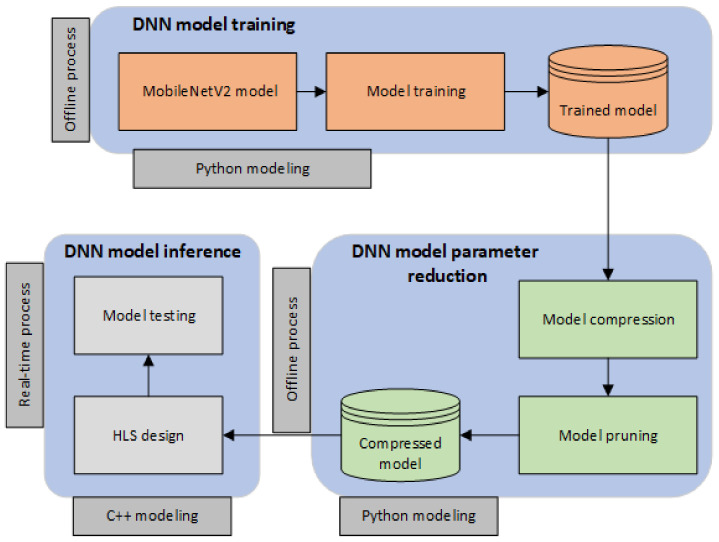
The design space exploration methodology we employ to validate our approach.

Figure 3 depicts the methodology under which we validate our approach. The evaluation of our designs is conducted through a mixture of offline and real-time operations which are implemented in Python and C++ languages. More specifically, the *DNN model training* is initially evoked, which utilizes the CIFAR-10 dataset to train the MobileNetV2 model. The training process is conducted offline, and it is implemented in Python programming language. In the sequel, the *DNN model parameter reduction* operation takes place, which performs model compression and model pruning on the trained DNN model in order to reduce the parameter volume. This process is also programming in a high-level language (Python), and it generates a compressed model, i.e., a DNN model with a lower amount of parameters. Finally, for the *DNN model inference*, we design the hardware using C++ and we test the compressed model on the accelerator, which is generated through the HLS operation. Notably, we apply our methodology after the DNN training is completed in both offline (*DNN model parameter reduction*) and real-time (*DNN model inference*) conditions.

### 5.3. Exploration Results

In this section, we present the results we obtained during the experimentation process. We illustrate data that depict the error rate, the model size reduction, the inference accuracy, the inference latency, the power efficiency and the resource utilization under several implementations. Furthermore, we provide comparisons with baseline solutions (BASE-50 and BASE-100) in order to obtain a clear picture of the performance of the designs.

#### 5.3.1. Pruning Error Rate

**Figure 4:**
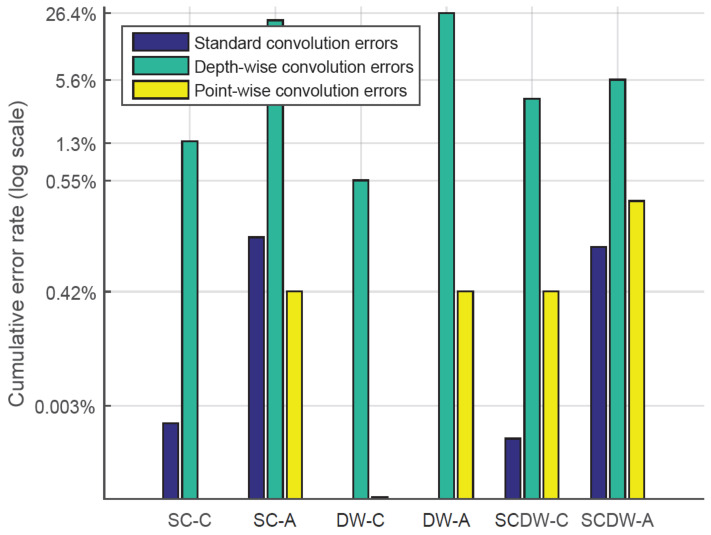
The observed error rate percentages for each layer type under different pruning configurations.

Figure 4 illustrates the percentage of error rate under different pruning configurations. Pruning errors occur since the process of removing model weights leads to information loss and thus introduces errors to the model. Within this context, the *cumulative error rate* is considered the sum of the model errors under a specific pruning configuration. It is expected that error-tolerant models are less likely to degrade their inference accuracy when high error rates are considered, whereas error-intolerant models will depict a higher degradation of their inference accuracy in the presence of high error rates.

The error rate is presented in logarithmic scale and the figure incorporates errors from different network layers, i.e., depth-wise, point-wise, and standard convolutional. The corresponding error rates are calculated over the baseline designs (*BASE-50* and *BASE-100*), which do not employ any pruning techniques, and thus, they are considered to have a 0% error rate. Below, we define the design acronyms used in the corresponding figure, as described in Table 2:

- SC-C: Standard convolution layers pruning, conservative approach.
- SC-A: Standard convolution layers pruning, aggressive approach.
- DW-C: Depth-wise convolution layers pruning, conservative approach.
- DW-A: Depth-wise convolution layers pruning, aggressive approach.
- SCDW-C: Standard and depth-wise convolution layers pruning, conservative approach.
- SCDW-A: Standard and depth-wise convolution layers pruning, aggressive approach.

Generally, conservative pruning techniques achieve fewer errors, depending on the layer types that are being pruned. For example, SC-C pruning results in a 0.002% error rate in SC layers, 1.36% in DW layers and 0.0003% in PW layers. This means that the *conservative pruning* of SC layers creates a small amount of errors that accumulate within the network, resulting in an error propagation phenomenon that extends to DW and PW layers as well. Despite this effect, the average error rate for *conservative pruning* techniques is 1.8% when taking into account the whole design space. Furthermore, specific conservative pruning approaches (such as DW-C) achieve no errors whatsoever in SC layers, thus preserving the network accuracy despite the information loss. On the contrary, *aggressive pruning* leads to an increased error rate, since weights are more easily eliminated as the cut-off threshold is set to a higher value. The average error rate for the *aggressive pruning* approach is 18.3%, which is higher compared to the *conservative pruning* technique. On the contrary, in case of SCDW-C and SCDW-A pruning techniques, the gap between *aggressive* and *conservative pruning* error rate narrows, since there, the average error rate difference is 2.41%. We deduce that the *aggressive pruning* of both SC and DW convolution layers may lead to better results, in terms of error rate, compared to the individual pruning of SC or DW layers only. The opposite applies for the *conservative pruning* approach, since the error rate of SCDW-C is higher compared to SC-C and DW-C techniques. We should also note that the clock frequency of the design or the network compression type (CSR or CSC) do not play any role to the overall error rate of the model and thus, such data are excluded from the figure.

#### 5.3.2. Model Parameter Reduction

**Figure 5:**
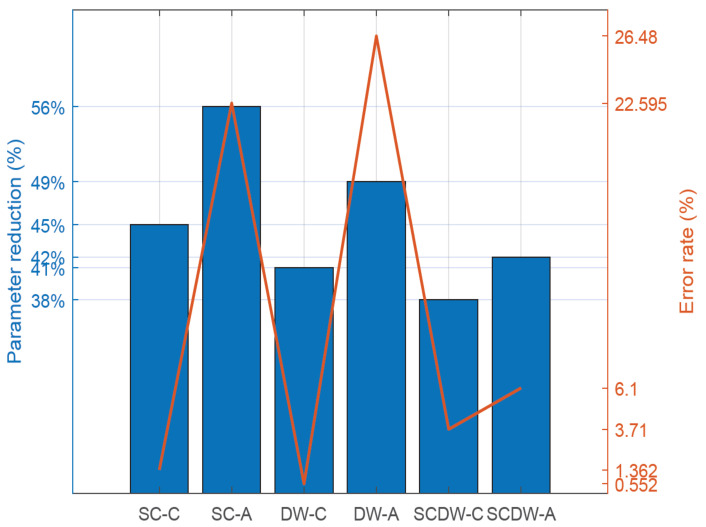
Comparison between network error rate and model parameter reduction under different pruning configurations.

In Figure 5, we depict the model parameter reduction along with the corresponding error rate for each pruning technique. The error rate of each technique is calculated as the sum of individual errors within the neural network, as discussed in Section 5.3.1 above, while the parameter reduction is measured over a baseline MobileNetV2. Furthermore, we should note that that the pruning techniques (such as SC-C, SC-A, DW-C, DW-A, SCDW-C and SCDW-A) are performed on a compressed neural network (CSR or CSC) during the inference (test) process in order to measure their efficiency in compressed space representations. The baseline designs for our comparison are the BASE-50 and BASE-100, which achieve an error rate of 0%. The baseline designs are error-free, since they compress the model and they preserve the weight values intact. On the contrary, pruning removes weights with small contribution to the model, and thus, it results in information loss.

The error rate of a model is defined as the average difference between the measured and the expected values of its parameters. As a result, when high error rates result in a large degradation of the model performance, the model is considered to be error-intolerant. On the contrary, models that depict greater resilience to high error rates are classified as error-tolerant. In any case, despite the fact that there is a correlation between error rates and model accuracy, such a connection cannot be translated into a one-to-one relationship. We explore the effect of the error rates to the MobileNetV2 performance in the Section 5.3.3 below.

We observe that there is a strong correlation between error rates and model size reduction, since the more the model size is reduced, the more errors are prone to appear. For example, by applying the SC-C technique, we manage to reduce the model size by a factor of 45%, and we obtain an error rate of 1.4%. Furthermore, by employing an *aggressive pruning* approach, such as SC-A, we shrink the model size by a larger margin (56%) while also raising the error rate (22.5%). In terms of efficiency, the DW-C and SC-C pruning methodologies achieve the best performance, since they maximize the size-to-error ratio. Conversely, DW-A and SC-A demonstrate lower performance as they impose heavy error penalties on the model. Conclusively, *conservative pruning* techniques reduce the model parameter count by 41.3% on average, while they impose a 1/8% error rate penalty to the designs. On the other hand, *aggressive pruning* approaches shrink the model by 49% on average, and they impose a 18.3% error penalty.

#### 5.3.3. Inference Accuracy

**Figure 6:**
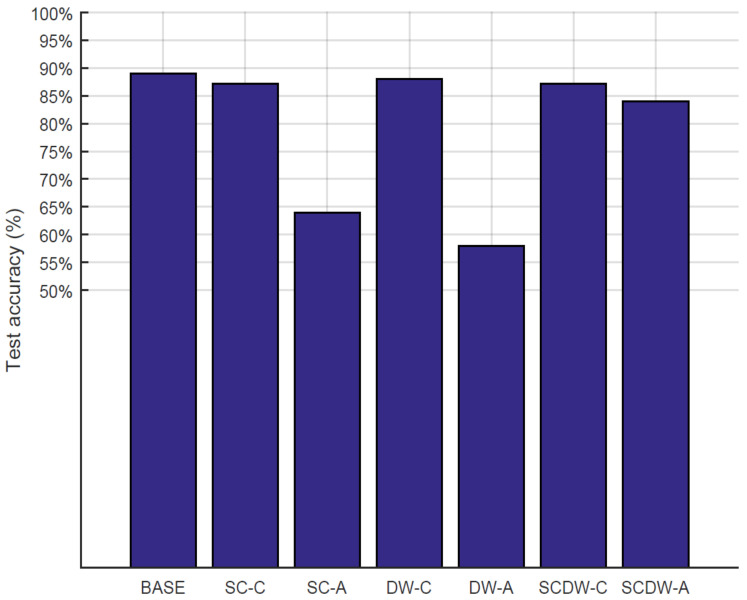
The test accuracy on the CIFAR-10 data set for several pruning techniques.

The performance of each adopted approach, in terms of inference (test) accuracy, is illustrated in Figure 6. The BASE representations achieve 89% accuracy, which is the highest among the designs, since their compression methods prevent information loss. Regarding the pruning methodologies, the best-performing approach is the DW-C (88% accuracy), while the worst performing is the DW-A (58%). Generally, the *conservative pruning* achieves better results as it manages to eliminate model parameters without affecting the overall test accuracy of the model. Regarding the *aggressive pruning* approaches, the SCDW-A is the best-performing design, since it achieves 84% accuracy.

There is a clear correlation between the error rate and the inference accuracy levels of the MobileNetV2 model, since higher error rates lead to lower accuracy results. However, the error rate by itself cannot provide us with adequate information on *how much* will the test accuracy will drop. Other parameters should also be considered such as the error rate per layer or the layer depth. Thus, the causal relationship between the network error rate and the model inference accuracy is expected to differentiate between individual DNN models.

Test accuracy can also be considered in conjunction with the model size, especially for the inference operation in low-power designs, where resource availability is constrained. Under this premise, the SC-C, followed by the SCDW-A, pruning are the best performing methods, since they manage to maintain high levels of accuracy while also contributing to a considerable amount of parameter reduction. From this point of view, the aggressive pruning approaches compensate for the accuracy loss by shrinking the model size by a large margin.

#### 5.3.4. Inference Latency

**Figure 7:**
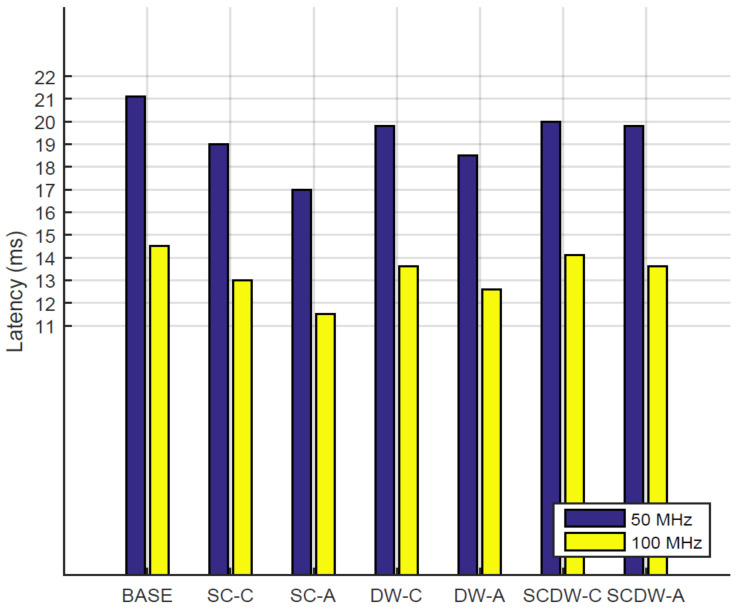
The inference latency of each pruning configuration, measured in milliseconds.

The inference latency refers to the amount of time required (in ms) to forward pass an input image through the network layers. Latency is a very important metric for the inference process since and indicates the real-time performance of a system, since it designates the amount of images a system can infer in a second. In this work, we utilize both 50 MHz and 100 MHz clock frequencies in order to measure the latency of our designs. Figure 7 depicts the evaluation results for each pruning configuration. Generally, designs that utilize a higher operating frequency (100 MHz) perform better (by 70% on average) compared to implementations that employ a 50 MHz clock. The baseline implementations depict the largest latency values (21.1 and 14.5 ms), since they also require more computations to complete. The fastest implementation is the SC-A, which requires 17 and 11.5 ms to perform an inference operation under 50 MHz and 100 MHz, correspondingly. Regarding the comparison with the baseline system, *conservative pruning* techniques reduce the inference latency by 7% under a 50 MHz clock and by 8% under a 100 MHz clock on average. The *aggressive pruning* methods achieve better speedup factors, since they perform 15% and 15% (for 50 MHz and 100 MHz, respectively) better compared with the baseline designs. From above, we can conclude that the clock frequency of the system is one of many parameters that affect its performance. There is no linear correlation between the increase of the clock frequency and the application performance, since there are a lot of parameters that affect the inference latency. Such parameters include but are not limited to BRAM access latency, BRAM read/write latency and the HLS accelerator–BRAM communication latency.

#### 5.3.5. Power Efficiency

The power efficiency, in terms of MOps/W, for baseline and CSR/C implementations is depicted in Table 3. CSR/C-50 design depicts the lowest power requirements (1.8 W), while the BASE-100 design requires the most power (2.1 W) among the tested implementations. This behavior is expected, since the power consumption of a system is highly dependent on the operating clock frequency and on the complexity of the circuit. To this end, we expect designs that operated within 100 MHz to depict higher power requirements compared with designs that employ a 50 MHz clock. Furthermore, model sparsification, compression and pruning optimizations reduce the computational intensity of the models, and thus, they minimize the complexity of control and arithmetic logic for the inference process. This observation is verified, since the power consumption of the CSR/C implementations is lower than BASE designs (4% for 50 MHz and 5% for 100 MHz implementations). Regarding the power efficiency of the systems, CSR/C-100 achieves the highest efficiency (500 MOps/W), and the BASE-50 depicts the lowest (322 MOps/W). Generally, CSR/C designs are more power efficient than their BASE counterparts, since they combine low power consumption and reduced computation complexity. More specifically, CSR/C-50 achieve 15% better power efficiency than BASE-50, while CSR/C-100 outperform the BASE-100 by 17.

#### 5.3.6. Resource Utilization

Table 4 depicts the FPGA resource utilization of BASE and CSR/C implementations. In terms of resource utilization, all the CSR/C implementations (i.e., SC-C, SC-A, DW-C, DW-A, SCDW-C, SCDW-A) present the same area requirements, and thus, we denote them as CSR/C-50 and CSR/C-100 correspondingly. This behavior is expected, since pruning operations lower the amount of DNN parameters and decrease the computational complexity of the models. On the contrary, hardware costs stay the same within different pruning configurations, because the pruned operations are executed on the specialized HLS accelerators that reserve a fixed amount of logic resources. We observe that both BASE and CSR/C designs utilize a small portion of the FPGA’s resources with the CSR/C implementations to consume the lowest amount of resources. The highest resource reservation ratio comes from the BRAM tiles, since the neural network requires a significant amount of storage space. Moreover, the LUT and register reservation rates are relatively low, and each one of them accounts for less than 40% of the available resources.

## 6. Conclusions

In this article, a design space exploration using a state-of-the-art HLS tool for sparse versions of MobileNetV2 was investigated. Experimental results prove that HLS tools can generate hardware designs that can be efficiently mapped into FPGAs directly from high-level languages, such as C/C++, without requiring long development cycles in the RTL level. The implementation of the proposed sparse MobileNetV2 has been made with sparse matrix storage and pruning techniques in the different convolution layers and can achieve high inference throughput. Finally, we have demonstrated that even with a high sparsity ratio, MobileNetV2 can achieve good accuracy in the CIFAR-10 data set, while the resource reservation rate and power utilization levels are significantly reduced in the targeted FPGA.

## Figures and Tables

**Table 1:** The MobileNetV2 model characteristics in terms of computational complexity.

Layer Type	Amount	Operations
Standard convolution layers	10	7 MFLOPs
Depth-wise convolution layers	9	3 MFLOPs
Point-wise convolution layers	9	3 MFLOPs
Total	28	13 MFLOPs

**Table 2:** HLS configuration and parameters of each implemented design.

Design Name	Compression Type	Frequency	Pruning Layers	Pruning Approach
BASE-50	None	50 MHz	None	None
CSR/C-50-SC-C	Compressed sparse row/column	50 MHz	Standard convolution	Conservative
CSR/C-50-SC-A	Compressed sparse row/column	50 MHz	Standard convolution	Aggressive
CSR/C-50-DW-C	Compressed sparse row/column	50 MHz	Depth-wise convolution	Conservative
CSR/C-50-DW-A	Compressed sparse row/column	50 MHz	Depth-wise convolution	Aggressive
CSR/C-50-SCDW-C	Compressed sparse row/column	50 MHz	Standard and Depth-wise convolution	Conservative
CSR/C-50-SCDW-A	Compressed sparse row/column	50 MHz	Standard and Depth-wise convolution	Aggressive
BASE-100	None	100 MHz	None	None
CSR/C-100-SC-C	Compressed sparse row/column	100 MHz	Standard convolution	Conservative
CSR/C-100-SC-A	Compressed sparse row/column	100 MHz	Standard convolution	Aggressive
CSR/C-100-DW-C	Compressed sparse row/column	100 MHz	Depth-wise convolution	Conservative
CSR/C-100-DW-A	Compressed sparse row/column	100 MHz	Depth-wise convolution	Aggressive
CSR/C-100-SCDW-C	Compressed sparse row/column	100 MHz	Standard and Depth-wise convolution	Conservative
CSR/C-100-SCDW-A	Compressed sparse row/column	100 MHz	Standard and Depth-wise convolution	Aggressive

**Table 3:** The power efficiency of the baseline and CSR/C designs for 50 MHz and 100 MHz operating frequencies.

	BASE-50	BASE-100	CSR/C-50	CSR/C-100
Static power consumption	0.162 W	0.174 W	0.152 W	0.157 W
Dynamic power consumption	1.75 W	1.93 W	1.68 W	1.84 W
Total power consumption	1.912 W	2.104 W	1.831 W	1.997 W
Mops/W	322	426	373	500

**Table 4:** The resource utilization of the BASE and CSR/C implementations.

	BASE-50	BASE-100	CSR/C-50	CSR/C-100
Slice LUTs	36.5%	36.4%	33.7%	33.5%
Slice Registers	23.4%	23.3%	21.9%	22.1%
Block RAM Tiles	48.2%	48.2%	45.7%	45.7%

## Abbreviations

The following abbreviations are used in this manuscript:

DNN	Deep Neural Network
NN	Neural Network
IoT	Internet of Things
SoC	System on Chip
GPU	Graphics Processing Unit
CPU	Central Processing Unit
DRAM	Dynamic Random Access Memory
MAC	Multiply and Accumulate
FPGA	Field-Programmable Gate Array
LUT	Look Up Table
MFLOPs	Million Floating Point Operations
HLS	High-Level Synthesis
SC	Standard Convolution
DW	Depth-Wise Convolution
PW	Point-Wise Convolution
SCDW	Standard and Depth-Wise Convolution
CSR	Compressed Sparse Row
CSC	Compressed Sparse Column
ms	Milliseconds
MOps	Mega-Operations
